# Gastrointestinal complications following on-pump cardiac surgery—A propensity matched analysis

**DOI:** 10.1371/journal.pone.0217874

**Published:** 2019-06-05

**Authors:** Katharina Marsoner, Andreas Voetsch, Christoph Lierzer, Gottfried H. Sodeck, Sonja Fruhwald, Otto Dapunt, Hans Joerg Mischinger, Peter Kornprat

**Affiliations:** 1 Department of General Surgery, Medical University of Graz, Graz, Austria; 2 Department of Cardiac Surgery, Medical University of Graz, Graz, Austria; 3 Department of Cardiac Surgery, Paracelsus Medical University, Salzburg, Austria; 4 Department of Emergency Medicine, Medical University of Vienna, Vienna, Austria; 5 Department of Anesthesiology and Intensive Care Medicine, Medical University of Graz, Graz, Austria; Azienda Ospedaliero Universitaria Careggi, ITALY

## Abstract

**Background:**

Gastrointestinal complications following on-pump cardiac surgery are orphan but serious risk factors for postoperative morbidity and mortality. We aimed to assess incidence, perioperative risk factors, treatment modalities and outcomes.

**Material and methods:**

A university medical center audit comprised 4883 consecutive patients (median age 69 [interquartile range IQR 60–76] years, 33% female, median logistic EuroScore 5 [IQR 3–11]) undergoing all types of cardiac surgery including surgery on the thoracic aorta; patients undergoing repair of congenital heart disease, implantation of assist devices or cardiac transplantation were excluded. Coronary artery disease was the leading indication for on-pump cardiac surgery (60%), patients undergoing cardiac surgery under urgency or emergency setting were included in analysis. We identified a total of 142 patients with gastrointestinal complications. To identify intra- and postoperative predictors for gastrointestinal complications, we applied a 1:1 propensity score matching procedure based on a logistic regression model.

**Results:**

Overall, 30-day mortality for the entire cohort was 5.4%; the incidence of gastrointestinal complications was 2.9% and median time to complication 8 days (IQR 4–12). Acute pancreatitis (n = 41), paralytic ileus (n = 14) and acute cholecystitis (n = 18) were the leading pathologies. Mesenteric ischemia and gastrointestinal bleeding accounted for 16 vs. 18 cases, respectively. While 72 patients (51%) could be managed conservatively, 27 patients required endoscopic/radiological (19%) or surgical intervention (43/142 patients, 30%); overall 30-day mortality was 12.1% (p<0.001). Propensity score matching identified prolonged skin-to-skin times (p = 0.026; Odds Ratio OR 1.003, 95% Confidence Interval CI 1.000–1.007) and extended on-pump periods (p = 0.010; OR 1.006, 95%CI 1.001–1.011) as significant perioperative risk factors.

**Comment:**

Prolonged skin-to-skin times and extended on-pump periods are important perioperative risk factors regardless of preoperative risk factors.

## Introduction

Gastrointestinal complications (GIC) following on-pump cardiac surgery are orphan but serious risk factors for postoperative morbidity and mortality [1–6]. The level of evidence regarding incidence, prevalence, perioperative and postoperative risk factors, treatment options, and outcomes is low, based on a few reports [1–10]. We aimed to provide a systematic and comprehensive report of our institutional 5-year experience in a consecutive series of 4883 patients undergoing on-pump cardiac surgery for all types of cardiac surgery except surgery of congenital heart disease, implantation of ventricular assist devices or cardiac transplantation. As we focused on identifying potentially modifiable perioperative risk factors, we applied a 1:1 propensity score matching (PSM) procedure based on a logistic regression model including all preoperative risk factors according to the EuroScore model [11].

## Materials and methods

We performed a university medical center audit between 2008 and 2013 comprising 4883 consecutive adult patients at the Department of Cardiac Surgery, Medical University of Graz, Austria, Europa, who had undergone on-pump-cardiac surgery for all types of cardiac surgery under elective, urgent or emergent setting, with baseline data according to the additive and logistic EuroScore I risk prediction tool. Peri- and postoperative patient data were entered in a prospective cardiac surgical database along with relevant clinical complications and 30-day follow-up data. Patients undergoing off-pump surgery or minimized extracorporeal circulation (MECC) surgery were excluded as well as patients undergoing surgery for congenital heart disease, implantation of ventricular assist devices or cardiac transplantation. Patients undergoing surgery of the thoracic aorta (with or without deep hypothermic circulatory arrest) were included in the study cohort. The dataset was 99% complete.

All patients who were seen by a consultant in visceral surgery were defined as having gastrointestinal complications regardless of the initial treatment they had undergone (medical treatment alone, endoscopic, interventional and/or surgical). Gastrointestinal complications were classified according to their frequency: (acalculous) cholecystitis, mechanical or paralytic (sub)ileus, primarily non-ischemic perforation, (sero)pancreatitis, intestinal ischemia, upper and lower gastrointestinal bleeding, and other rare gastrointestinal complications.

Paralytic ileus or subileus was defined to include all patients in whom gastrointestinal motility could not be recovered with standard procedures such as early mobilization, sufficient hydration, correction of serum electrolytes as well as perioperative administration of domperidon and metoclopramid, and oral and rectal laxatives. In proven absence of mechanical obstruction, intravenous neostigmine as well as oral erythromycin was administered to regain gastrointestinal motility; if appropriate, the colon was decompressed.

Postoperative (sero)pancreatitis by definition included all patients who presented elevated leucocyte and CRP counts as well as serum lipase and amylase levels, regardless of the presence of abdominal pain. All underwent routine abdominal computed tomography.

Diagnosis and selection of treatment modalities including invasive interventions (endoscopic and surgical) were made in accordance with standard procedures.

All cardiac-related procedures were performed on-pump according to standard anesthetic and surgical techniques: in brief, after median partial or full sternotomy and heparinization, cardiopulmonary bypass was established via ascending aortic and right atrial or bicaval cannulation; in selected cases, other cannulation techniques via the femoral or axillary approach were applied. Cardioplegic arrest was maintained via cold blood cardioplegia in antegrade or retrograde fashion as appropriate. Complex cardiac surgical procedures including aortic root surgery were performed in moderate or deep hypothermia; in addition, deep hypothermic circulatory arrest was chosen for aortic arch surgery in all cases. Heparinization was antagonized via intravenous protamine administration under ACT (activated clotting time) monitoring after weaning from extracorporeal circulation. Patients were extubated immediately in the absence of adverse signs. According to institutional policy, all patients received proton pump inhibitors for ulcer prophylaxis and oral domperidone or intravenous metoclopramide to prevent perioperative gastrointestinal atony.

Routine preoperative gastroscopy was performed to reduce the risk of perioperative upper gastrointestinal bleeding; with erosive gastritis or peptic ulcer, elective cardiac surgery was postponed until endoscopy verified healing. Patients with longer ventilation periods all received enteral nutrition as early as possible. Mobilization was started on postoperative day one when patients were extubated and hemodynamically stable and/or transferred to the normal cardiac surgical ward.

Due to the retrospective nature of this study and no study-specific interventions and/or examinations, the ethical committee of the medical university of Graz waived the need for patient consent.

### Statistical analysis

Continuous data are presented as the median and the interquartile range (IQR; 25th to 75th percentile). Discrete data are given as counts and percentages. For baseline comparisons, the χ2 test for categorical variables and Mann-Whitney U test for continuous variables were used for univariate analysis.

Since a standard statistical approach could not be pursued due to group size differences at baseline, a propensity matched (PSM) study was designed for the occurrence of gastrointestinal complications: First, all covariates of the original EUROSCORE were forced into a binary logistic regression model to calculate individual propensity scores. Model-fit and regression diagnostics followed standard procedures. Then, a 1:1 matching was performed via an adapted PSM Macro by Raynald Levesque (modified for use with propensity matching by John Painter) for SPSS [12] (http://faculty.umb.edu/william_holmes/clarkmacro.htm; last access 01.05.2017) with a maximum caliper of 0.1. Following PSM, a univariate logistic regression analysis was employed to obtain odds ratios (OR) and their respective 95% confidence intervals (95% CI).

A two-sided p-value <0.05 was considered statistically significant and SPSS 24.0 for Windows (IBM Inc, Somers, NY, USA) was used for all statistical analyses.

## Results

**Fig 1:**
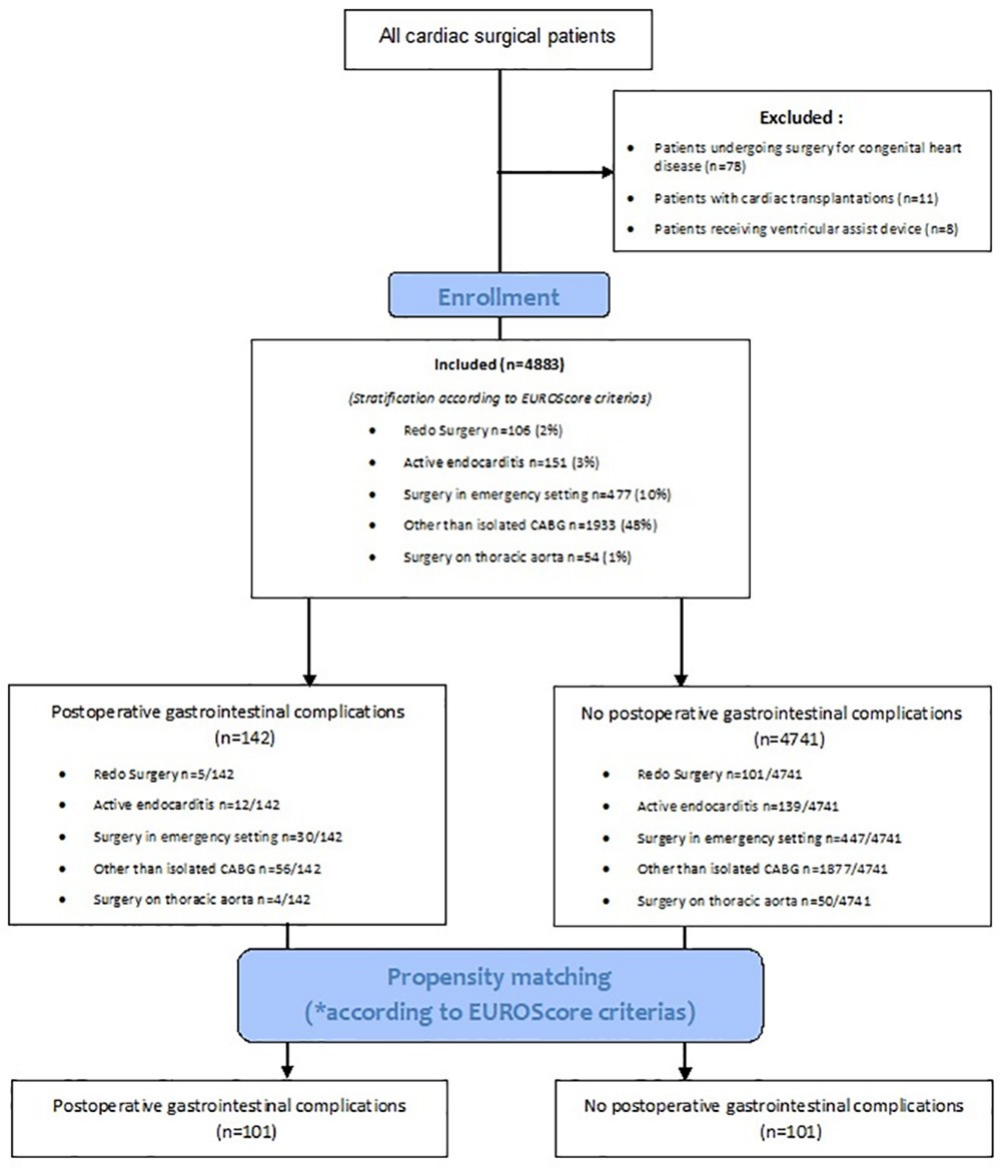
Study flow chart. CABG Coronary artery bypass grafting.

Between 2008 and 2013, 4883 consecutive patients underwent on-pump cardiac surgery at our institution (median age 69 [interquartile range IQR 60–76] years, 33% female sex, median logistic EuroScore [ES] 5 [IQR 3–11]). Coronary artery and aortic valve disease were the leading indications for surgery (60 and 40%, respectively). Patient’s selection according to EuroScore criterias is presented in Fig 1.

We identified a total of 142 patients with GIC requiring medical, interventional or surgical support (median age 70, IQR 60–76 years, 26% female sex, median logistic EuroScore [ES] 9 [IQR 4–20]. For details see S1 and S2 Tables.

Besides significant differences in preoperative risks according to the EuroScore model in the unmatched cohorts at baseline, prolonged skin-to-skin times (250 (IQR 199–319) vs. 225 (IQR 182–274) minutes; p<0.0001; OR 1.003, 95% CI 1.002–1.004) and extended on-pump periods (135 (IQR 96–176) vs. 115 (IQR 90–146) minutes; p<0.0001; OR 1.004; 95% CI 1.002–1.0007) were significant perioperative risk factors; multi-organ failure (24 vs. 3%; p<0.0001; OR 12.42; 95% CI 8.119–19.067), acute renal failure (21 vs. 3%; p<0.0001; OR 7.719, 95% CI 5.007–11.900) and pneumonia (15 vs. 2%; p<0.0001; OR 7.375, 95% CI 4.514–12.042) were postoperative risk factors. The type of surgery (p = 0.88; OR 1.028; 95% CI 0.711–1.488) obviously did not play a significant role in the occurrence of GIC. For details see S3 and S4 Tables.

The overall cumulative incidence of postoperative GIC was 2.9%, corresponding to a prevalence of 5.8 cases per 1000 on-pump surgeries per year.

**Fig 2:**
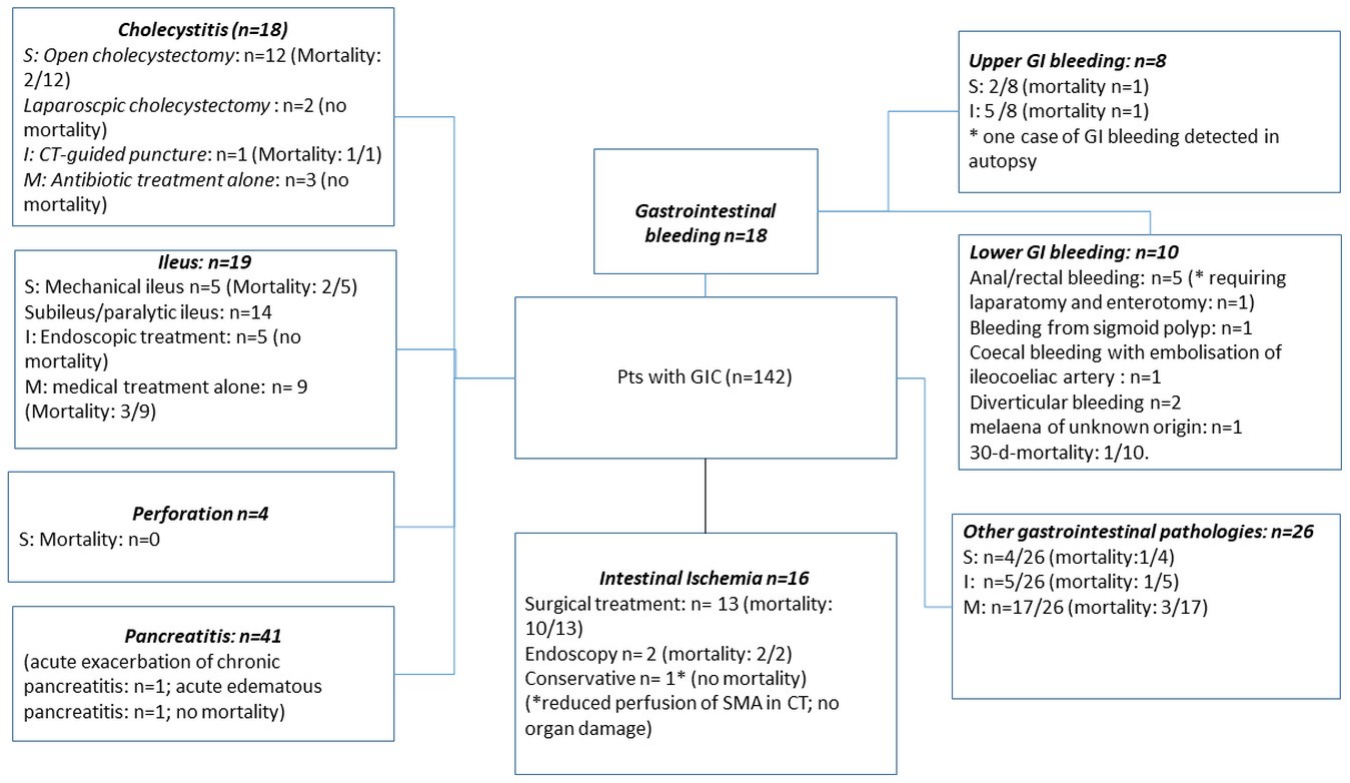
Treatment and outcome details of all patients with gastrointestinal complications. S surgical intervention, I interventional treatment, M medical treatment, CT computed tomography, GIC gastrointestinal complication, SMA superior mesenteric artery, GI gastrointestinal.

Median time to complication was eight days (IQR 4–12). Acute pancreatitis (41 patients), paralytic ileus (14 patients) and acute cholecystitis (18 patients) were identified as leading pathologies. Mesenteric ischemia and gastrointestinal bleeding accounted for 16 and 18 cases, respectively (11 vs. 13%). A conservative medical treatment approach could be followed in 72 patients (51%), whereas 27 patients required radiological or endoscopic (19%) or surgical intervention (n = 43; 30%). Details on incidence, prevalence, time to complication and mortality are presented in Tables 1 and 2; treatment and outcome details of patients with GIC are presented in Fig 2.

**Table 1:** Details about GIC.

*Type of GIC*^a^	*Incidence*	*Prevalence*	*Time to complication(days; median; IQR*^b^*)*	*Mortality*
Overall	2.9%	0.26%	8 (4–12)	23%
Cholecystitis	0.37%	0.025%	10 (8–18)	17%
Ileus	0.39%	0.028%	4 (3–6)	26%
Perforation	0.08%	0.02%	10 (7–35)	None
Pancreatitis	0.84%	0.02%	9 (6–12)	None
Ischemia	0.33%	0.08%	5 (4–8)	75%
GI bleeding	0.18%	0.013%	13 (2–15)	22%
Other GIC	0.53%	0.025%	7 (4–13)	19%

^a^GIC gastrointestinal complication.

^b^IQR interquartile range.

**Table 2:** Other gastrointestinal complications in detail.

*Other GIC*^a^ *pathologies (n = 26)*	n
Hepatic failure	7
Gall bladder hydrops	6
Diarrhea	4
Severe gastric paresis	1
Ogilvie syndrome	1
Abdominal compartment syndrome	1
Stenosing sigmoid diverticulitis	1
Polytrauma with posttraumatic aortic pseudoaneurysm, two-stage liver rupture	1
Acute abdomen of unknown origin	1
PEG^a^ tube insertion	1
Intraabdominal bleeding (caused by laceration of right liver lobe)	1
Stenosing rectal carcinoma	1

^a^PEG Percutaneous endoscopic gastrostomy

Overall 30-day mortality of the unmatched cohort was 5.4%, for patients with GIC 23% (p<0.001).

Open cholecystectomy was performed in 12 of 18 patients with acute cholecystitis; two patients who were sufficiently hemodynamically stable to tolerate pneumoperitoneum underwent laparoscopic cholecystectomy. In three cases, antibiotic treatment alone sufficed to cure the cholecystitis. One patient underwent CT-guided puncture; two patients underwent bedside ultrasound guided cholecystotomy as bridging to cholecystectomy until they were stable enough for abdominal surgery.

Five patients in all underwent abdominal surgery for mechanical ileus: in three cases, intra-abdominal adhesions from preceding abdominal surgery caused mechanical ileus; one patient presented with incarceration of an inguinal hernia, and one with incarceration of an incisional hernia.

All patients with gastrointestinal perforation underwent surgery immediately. One case was an iatrogenic gastric perforation after insertion of a percutaneous gastroenterostomy (PEG) tube and one patient presented with perforated sigmoid diverticulitis; the two remaining patients had non-ischemic colonic perforations.

In the upper GI bleeding group, most patients were bleeding from a gastric or duodenal ulcer; in four cases, the origin of bleeding could not be identified. Lower GI bleeding was mostly caused by anal or rectal bleeding from pre-existing hemorrhoids; in one case there was bleeding from sigmoid polyp. In three cases, CT angiography showed diverticular bleeding, and one patient underwent embolization of the ileocolic artery.

Thirteen out of 16 patients underwent emergent abdominal surgery, in two cases with only bedside colonoscopy as both patients were in multi-organ failure and not fit for transfer to surgery or angiography. One patient had very high serum lactate without clinical signs of acute abdomen. CT angiography indicated reduced perfusion in the superior mesenteric artery territory but no end-organ damage; as there were no clinical or radiological signs of enteric damage and serum lactate values quickly normalized, a watchful waiting strategy was pursued and the patient could be discharged to cardiologic rehabilitation on postoperative day 14.

A propensity matched (PSM) analysis approach was then undertaken: First, all individual covariates of the original EUROSCORE were forced into a binary logistic regression model to calculate individual propensity scores. Then, a 1:1 match was performed (max. caliper 0.1), obtaining perfectly matched cohorts of 101 patients each. As given in Tables 3 and 4, there were no significant differences at perioperative baseline after matching.

**Table 3:** Baseline data according to EuroScore parameters (matched cohorts).

*Matched cohorts Baseline demographics*	*Pts with GIC*^a^ *(n = 101)*	*Pts without GIC*^a^ *(n = 101)*	*Two-sided p-value*
Age > 60 years	47 (47%)	47 (47%)	1.00
Female gender	25 (25%)	28 (28%)	0.75
Chronic pulmonary disease	28 (28%)	26 (26%)	0.87
Extracardiac arteriopathy	31 (31%)	35 (35%)	0.65
Neurological dysfunction	12 (12%)	11 (11%)	1.00
Previous cardiac surgery	3 (3%)	3 (3%)	1.00
Serum creatinine > 200 μmol/l	29 (29%)	32 (32%)	0.76
Active endocarditis	9 (9%)	10 (10%)	1.00
Critical preoperative state	0	0	1.00
Unstable angina	23 (23%)	24 (24%)	1.00
LV^b^ function < 30%	31 (31%)	36 (36%)	0.55
Recent myocardial infarction	37 (37%)	37 (37%)	1.00
Pulmonary hypertension > 60 mm HG	4 (4%)	4 (4%)	1.00

^a^GIC Gastrointestinal complication

^b^LV left ventricular

**Table 4:** Surgical risk profile (matched cohorts).

*Matched cohortsSurgical risk profile*	*Pts with GIC*^a^ *(n = 101)(median/IQR*^b^*)*	*Pts without GIC*^a^ *(n = 101)(median/IQR*^b^*)*	*Two-sided p-value*
Emergency	21 (21%)	17 (17%)	0.59
Other than isolated CABG^c^	46 (46%)	47 (47%)	0.89
Surgery on thoracic aorta	2 (2%)	0	0.50
Postinfarct septal rupture	0	0	1.00
Additive EuroSCORE	7 (4–11)	7 (4–11)	0.98
Logistic EuroSCORE	8 (3–23)	6 (3–20)	0.70

^a^GIC Gastrointestinal complication

^b^IQR interquartile range

^c^CABG coronary artery bypass grafting

Coronary artery and aortic valve disease were still the leading indications for surgery (56 vs. 55% and 27 vs. 34%, respectively) [Table 5].

**Table 5:** Surgical details (matched cohorts).

*Matched cohortsIntraoperative details*	*Pts with GIC*^*C*^ *(n = 101) n(%)*, *median (IQR*^b^*)*	*Pts without GIC*^a^ *(n = 101) n(%)*, *median (IQR*^b^*)*	*Two-sided p-value*
Skin-to-skin time (minutes)	240 (192–308)	210 (181–260)	0.016
Cardiopulmonary bypass time (minutes)	121 (96–173)	109 (89–135)	0.014
Aortic cross clamp time (minutes)	78 (58–116)	69 (57–89)	0.08
Deep hypothermia	4 (4%)	0	0.12
Intra-aortic ballon pump	13 (13%)	6 (6%)	0.15
***Type of surgery***			
Coronary bypass surgery	56 (56%)	55 (55%)	1.00
Aortic valve surgery	27 (27%)	34 (34%)	0.36
Mitral valve surgery	16 (16%)	15 (15%)	1.00
Tricuspid valve surgery	4 (4%)	5 (5%)	1.00
Surgery on ascending aorta	10 (10%)	6 (6%)	0.44
Surgery on aortic arch	2 (2%)	0	0.50

^a^GIC gastrointestinal complication

^b^IQR interquartile range

In the matched cohorts, prolonged skin-to-skin times (240 (192–308) vs. 210 (181–260) minutes; p = 0.026; OR 1.003, 95% CI 1.000–1.007) and extended on-pump periods (121 (966–173) vs. 109 (89–135) minutes; p = 0.010; OR 1.006, 95% CI 1.001–1.011) could be still identified as significant perioperative risk factors [Table 5], and multi-organ failure (p = 0.004; OR 3.74, 95%CI 1.518–9.213), acute renal failure (p = 0.012; OR 3.052, 95% CI 1.282–7.265) and pneumonia (p = 0.035; OR 2.718, 95% CI 1.074–6.875) as postoperative risk factors in univariate analysis. Type of surgery (p = 0.846; OR 0.943, 95% CI 0.542–1.642) as well as new onset of atrial fibrillation (p = 0.068; OR 1.762, 95% CI 0.959–3.263) remained non-significant [Table 6].

**Table 6:** Postoperative details (matched cohorts).

*Matched cohorts Postoperative details*	*Pts with GIC*^a^ *(n = 101) n(%)*	*Pts without GIC*^a^ *(n = 101) n(%)*	*Two-sided p-value*
Perioperative myocardial infarction	1 (1%)	3 (3%)	0.62
Postoperative circulatory arrest	2 (2%)	2 (2%)	1.00
Re-do	4 (4%)	5 (5%)	1.00
Revision for bleeding	9 (9%)	9 (9%)	1.00
Acute renal failure dependent upon dialysis	21 (21%)	8 (8%)	0.015
Pneumonia	17 (17%)	7 (7%)	0.05
Sepsis	9 (9%)	5 (5%)	0.41
Deep sternal wound infection	2 (2%)	2 (2%)	1.00
Multi-organ failure	22 (22%)	7 (7%)	0.004
30-d-mortality	21 (21%)	5 (5%)	0.003
Lost to follow-up	1 (1%)	1 (1%)	1.00

^a^GIC gastrointestinal complication

## Discussion

Gastrointestinal complications (GIC) following on-pump cardiac surgery are regarded as orphan but serious risk factors for postoperative morbidity and mortality [1–10]. Our comprehensive university medical center audit of 4883 consecutive adult patients revealed an overall incidence of GIC of 2.9% and a median time to complication of 8 days; 30-day mortality for patients with GIC was 23%.

GIC incidence following on-pump cardiac surgery has increased in recent decades, ranging from 0.5 to 4.17 percent in published studies [1–10] as increasing numbers of multi-morbid patients undergo more, and more complex cardiac surgical procedures [1]. In line with other publications we also saw a strong correlation between preoperative Euro Score I-values and GIC [4]. As there is still no uniform definition and standard of reporting GIC incidence [1–10], we sought to overcome this obvious bias and unlike others, we included all patients who were seen by a consultant visceral surgeon regardless of the treatment modality.

Acute pancreatitis, paralytic ileus and acute cholecystitis were identified as the leading pathologies.

The incidence of postoperative pancreatitis varies among previous reports due to lack of a uniform definition: in our study, all patients with pathologic pancreatic lipase levels (> 300 units/liter) and positive laboratory signs of inflammation were classified as having this particular complication. Interestingly, only two patients showed obvious clinical and radiologic signs of disease. Adapted diet, oral pancreatic enzyme supplementation, and in severe cases, intravenous somatostatin, were applied successfully in all these patients [13–15].

Postoperative intestinal paralysis is regarded as a common complication of heart surgery [1; 6]. Immobility, administration of high doses of opioid drugs and delayed or absent enteral alimentation can potentiate the adverse effects of intraoperative mucosal damage caused by on-pump surgery. In our cohort, all cases of paralytic ileus could be managed with endoscopic or medical therapy. Nevertheless, the postoperative mortality in this particular subgroup was excessive at 26% due to multi-organ failure [16–20].

Acute cholecystitis has been reported to be one of the most frequent GICs [1; 3; 4; 6] and we found that the majority of patients do present with typical clinical (fever, right upper abdominal pain, positive Courvoisier sign), sonographic and laboratory signs of disease. Interestingly, only two patients had pre-existing asymptomatic cholecystolithiasis. Uncomplicated cases of postoperative cholecystitis could be managed with antibiotic treatment only [21]; in other cases laparoscopic cholecystectomy or open cholecystectomy were the treatments of choice, with percutaneous cholecystostomia as a definitive or bridge therapy in only two cases.

Mesenteric ischemia, whether occlusive or non-occlusive, is regarded as the most fatal GIC following cardiac surgery. Like other authors, we observed excessive mortality of 75% in this particular subgroup. Due to the absence of typical and clear clinical signs or masking by signs of other, more common complications, mesenteric ischemia demands early clinical suspicion and diagnosis to secure successful outcomes [3; 22; 23].

Several authors could clearly demonstrate that increased pre-operative morbidity does strongly correlate with the incidence and mortality of postoperative gastrointestinal complications [1–10]. As the risk factors associated there are not modifiable, our university audit focused on intra- and postoperative risk factors that have not yet been defined. Unlike others, we failed to identify either atrial fibrillation or intra- or postoperative mechanical support with the intra-aortic balloon pump (IABP) as significant risk factors in this cohort [1; 3]. Perhaps because the broad benefits of IABP have not been completely validated, its use is rather limited in our cohort; further, growing awareness of atrial fibrillation as a risk factor has encouraged application of preventive measures [24].

In our comprehensive and contemporary university center audit, however, we could clearly observe that overall skin-to-skin times as well as on-pump periods were significantly higher in patients with postoperative GIC, regardless of the preoperative risk profile.

These particular findings can be explained by reduced splanchnic perfusion during extracorporeal circulation periods with compromised mucosal integrity and development of gastrointestinal pathologies [16–20].

Although intra-operative complications that may prolong surgery and on-pump time cannot be foreseen, such patients are at risk and should be watched closely for any sign of GIC during the postoperative period. We further find that close observation should be extended to patients who are at additional risk with postoperative acute renal failure, pneumonia and multi-organ failure.

Finally, patients scheduled for complex cardiac surgery should undergo meticulous evaluation of operative strategy in advance to limit on-pump time periods as a measure with potential to limit the risk for GIC complications after on-pump cardiac surgery.

### Limitations

All the limitations of a retrospective study design have to be acknowledged for our study; these are not limited to individual patient profiles, management in the operating room and postoperative care. This study does, however, cover a large cohort of consecutive patients within a limited and short timeframe that decreases the potential negative impact of the retrospective design on our conclusions. Secondly, we have applied a propensity matched study design to account for differences at baseline to create identical study cohorts in order to clearly identify peri- and postoperative risk factors. Matching was based on the original EuroScore I model as the cardiac database was launched prior the introduction of the newer EuroScore II model. As we did not focus on the predictive power of the EuroScore for the incidence of gastrointestinal complications following on-pump surgery, this limitation has to be considered as negligible. Finally, we did not consider the STS score or other scores for risk profiling as they are scarely used in clinical routine in Europe, therefore generalizability of our conclusions to other setting might be limited.

### Conclusion

Incidence and prevalence of GIC following on-pump cardiac surgery are low but associated with a high rate of morbidity and mortality. Close surveillance, prompt diagnosis and adequate interdisciplinary treatment of GIC are the keys to success. Further work should focus on preventative strategies for GIC following on-pump cardiac surgery.

## Supporting information

S1 Table Baseline data. Unmatched cohort, preoperative patient’s characteristics according to EuroScore parameters.
(DOCX)

S2 Table Surgical risk profile. Unmatched cohort.
(DOCX)

S3 Table Intraoperative details. Unmatched cohort.
(DOCX)

S4 Table Postoperative details. Unmatched cohort.
(DOCX)
