# Supplementary material for: Gastrointestinal complications following on-pump cardiac surgery—A propensity matched analysis
Source: PLoS One. 2019 Jun 5;14(6):e0217874. doi: 10.1371/journal.pone.0217874 (PMC6550404; doi:10.1371/journal.pone.0217874)
Supplement: S1 Table — Unmatched cohort, preoperative patient’s characteristics according to EuroScore parameters. (DOCX) [file pone.0217874.s001.docx]

| ***Unmatched cohorts***  ***Intraoperative details*** | ***Overall cohort (n=4883)***  ***n (%),median (IQR^a^)*** | ***pts with GIC^b^ (n=142); n (%);***  ***median (IQR^a^)*** | ***pts without GIC^b^ (n=4741); median (IQR^a^)*** |
| --- | --- | --- | --- |
| Skin-to-skin time (minutes) | 226 (182 – 275) | 250 (199 – 319) | 225 (182 – 274) |
| Cardiopulmonary bypass time (minutes) | 115 (90 – 147) | 135 (96 – 176) | 115 (90 – 146) |
| Aortic cross clamp time (minutes) | 75 (58 – 100) | 86 (58 – 125) | 75 (58 – 99) |
| Deep hypothermia | 57 (1%) | 5 (4%) | 52 (1%) |
| Intra-aortic ballon pump | 220 (5%) | 18 (13%) | 202 (4%) |
|  |  |  |  |
| ***Type of surgery*** |  |  |  |
| Coronary bypass surgery | 2950 (60%) | 86 (61%) | 2864 (60%) |
| Aortic valve surgery | 1930 (40%) | 49 (35%) | 1881 (40%) |
| Mitral valve surgery | 555 (11%) | 23 (16%) | 532 (11%) |
| Tricuspid valve surgery | 180 (4%) | 6 (4%) | 174 (4%) |
| Surgery on ascending aorta | 327 (7%) | 17 (12%) | 310 (7%) |
| Surgery on aortic arch | 54 (1%) | 4 (3%) | 50 (1%) |

S3 Table

a IQR interquartile range

b GIC gastrointestinal complication
