# Supplementary material for: Gastrointestinal complications following on-pump cardiac surgery—A propensity matched analysis
Source: PLoS One. 2019 Jun 5;14(6):e0217874. doi: 10.1371/journal.pone.0217874 (PMC6550404; doi:10.1371/journal.pone.0217874)
Supplement: S2 Table — Unmatched cohort. (DOCX) [file pone.0217874.s002.docx]

| ***Unmatched cohorts***  ***Surgical risk profile*** | ***Overall cohort (n=4883)***  ***n (%),median (IQR^a^)*** | ***pts with GIC^b^ (n=142); n (%);***  ***median (IQR^a^)*** | ***pts without GIC^b^ (n=4741); median (IQR^b^)*** |
| --- | --- | --- | --- |
| Emergency | 477 (10%) | 30 (21%) | 447 (9%) |
| Other than isolated CABG^c^ | 1933 (48%) | 56 (48%) | 1877 (48%) |
| Surgery on thoracic aorta | 54 (1%) | 4 (3%) | 50 (1%) |
| Postinfarct septal rupture | 1 (0.1%) | 0 | 1 (0.1%) |
| Additive EuroSCORE | 6 (4 – 9) | 8 (5 – 11) | 6 (4 – 9) |
| Logistic EuroSCORE | 5 (3 – 11) | 9 (4 – 20) | 5 (3 – 11) |

S2 Table

a IQR interquartile range

b GIC gastrointestinal complication

c CABG coronary artery bypass grafting
