# Supplementary material for: Gastrointestinal complications following on-pump cardiac surgery—A propensity matched analysis
Source: PLoS One. 2019 Jun 5;14(6):e0217874. doi: 10.1371/journal.pone.0217874 (PMC6550404; doi:10.1371/journal.pone.0217874)
Supplement: S4 Table — Unmatched cohort. (DOCX) [file pone.0217874.s004.docx]

| ***Unmatched cohorts***  ***Postoperative details*** | ***Overall cohort (n=4883)***  ***n (%)*** | ***pts with GIC^a^ (n=142); n (%)*** | ***pts without GIC^a^ (n=4741) n(%)*** |
| --- | --- | --- | --- |
| Perioperative myocardial infarction | 52 (1%) | 2 (1%) | 50 (1%) |
| Postoperative circulatory arrest | 90 (2%) | 6 (4%) | 84 (2%) |
| Redo | 130 (3%) | 9 (6%) | 121 (3%) |
| Revision for bleeding | 273 (6%) | 16 (11%) | 257 (5%) |
| Acute renal failure dependent upon dialysis | 189 (4%) | 30 (21%) | 159 (3%) |
| Pneumonia | 137 (3%) | 22 (15%) | 115 (2%) |
| Sepsis | 69 (1%) | 13 (9%) | 56 (1%) |
| Deep sternal wound infection | 29 (1%) | 2 (1%) | 27 (1%) |
| Multi-organ failure | 151 (3%) | 34 (24%) | 117 (3%) |
| 30-d-mortality | 260 (5%) | 32 (23%) | 228 (5%) |

S4 Table

a gastrointestinal complication
